# Application effect of BOPPPS teaching model on fundamentals of nursing education: a meta-analysis of randomized controlled studies

**DOI:** 10.3389/fmed.2024.1319711

**Published:** 2024-05-09

**Authors:** Yue Li, Xiao Li, Yan Liu, Yang Li

**Affiliations:** Nursing College of Yunnan University of Traditional Chinese Medicine, Kunming City, China

**Keywords:** BOPPPS (bridge-in), teaching model, ‘‘Fundamentals of Nursing’’ education, meta-analysis, GRADE quality evaluation

## Abstract

**Background:**

BOPPPS (bridge-in, learning objective, pretest, participatory learning, posttest, and summary) is a student-centered, closed-loop teaching model that emphasizes real-time communication and feedback.

**Objectives:**

The purpose of this study was to review and evaluate the effect of BOPPPS teaching model in “Fundamentals of Nursing” teaching.

**Methods:**

We conducted a meta-analysis of randomized controlled trials (RCTs) based on the BOPPPS teaching model in “Fundamentals of Nursing” teaching. To review domestic and foreign databases for the period 2010 to September 2023. Finally, 13 RCTs were included and the teaching outcomes were measured and analyzed. Two researchers independently identified, selected, and extracted data from the study and examined the risk of bias. The primary outcomes were students’ examination scores (theoretical scores: scores obtained in the nursing fundamentals course, reflecting students’ understanding and mastery of the course content; practical scores: assessment results based on practical application or experimental skills, evaluating students’ practical skill level). The secondary outcomes were self-learning ability score: indicators assessing students’ self-directed learning ability, reflecting their competence in independent learning and autonomous exploration; and satisfaction rate of teaching effect: the overall satisfaction rate of students with the teaching effects experienced during teaching process reflects the proportion of students’ acceptance and satisfaction with the teaching program. The results were evaluated using the Grading of Recommendations, Assessment, Development, and Evaluations (GRADE) profiler software. The GRADE profiler software is used to assess and grade the recommendations according to the GRADE (Grading of Recommendations Assessment, Development, and Assessment) criteria.

**Results:**

A total of 13 studies were included, consisting of 2,991 nursing students. Among them, 1,465 students were in the BOPPPS teaching group, while 1,526 students were in the traditional teaching group. The summary analysis of the main outcomes showed that the BOPPPS teaching model had significantly higher scores in theoretical score (MD = 3.35, 95% CI: 2.35–4.35, *Z* = 6.56, *p* < 0.00001), practice score (MD = 4.50, 95% CI: 1.95–7.05, *Z* = 3.45, *p* = 0.0006), and self-learning ability score (MD = 6.76, 95% CI: 5.38–8.14, *Z* = 9.60, *p* < 0.00001) compared to the traditional teaching group. The satisfaction rate of students in the BOPPPS teaching group regarding teaching effectiveness was 89% (95% CI = 0.84–0.93). The differences were statistically significant (*p* < 0.05). The GRADE evidence level for theoretical score and satisfaction rate of teaching effect is low. The evidence level for practice score is very low, and for self-learning ability score is moderate.

**Conclusion:**

The BOPPPS teaching mode is helpful to improve the theoretical score, practice score, and self-learning ability score of “Fundamentals of Nursing,” and improve the satisfaction rate of students to the teaching effect. The teaching effect is better than the traditional teaching method.

## Introduction

The goal of nursing education is to cultivate comprehensive and applied talents with diversified abilities (1). With the development of societal disciplines, there is an increasing demand for higher-quality nursing professionals. Therefore, it is particularly important to focus on how to cultivate high-quality nursing professionals. “Fundamentals of Nursing” is one of the most basic and important core courses in the curriculum system of nursing, and it is the basis of all professional courses (2). This course aims to not only develop students’ basic nursing knowledge and skills but also emphasize the cultivation of their abilities in problem-solving, critical thinking, and judgment (3). The goal is to enable students to apply fundamental theories and skills flexibly in clinical nursing, playing a crucial role in nursing education and teaching. The course “Fundamentals of Nursing” covers a wide range of content and requires students to have a high level of theoretical learning and practical skills. However, compared to other disciplines, the content of this course is more abstract and the knowledge points are more complex, making it challenging for students to learn (4).

Traditional nursing education often relies on a teacher-centered approach, where students passively receive knowledge, resulting in a lack of teacher-student interaction (5). This teaching method hinders students’ ability to think critically and fails to stimulate their independent learning capabilities. Most students lack critical thinking skills during their learning process and fail to integrate theoretical knowledge with practical knowledge through real-world clinical case studies. As a result, when they enter the clinical practicum, they often realize that they have not truly understood many of the procedures taught in the classroom and struggle to adapt to the demands of clinical nursing work (6). In recent years, although medical schools have gradually adopted diversified teaching methods (7, 8), there are still some problems, such as the large amount of information in classroom lectures and the lack of interaction between teachers and students, which cannot effectively solve the problem of the disconnect between theory and clinical practice in teaching (9, 10). As nursing is a practice-oriented field within medicine, it requires a combination of theoretical knowledge and clinical skills. In nursing education, deficiencies in the teaching and learning process, lack of perceived professional support, and disparities between simulated and real clinical practice can hinder students from effectively applying theoretical knowledge in clinical settings (11). Nursing educators have recognized the gap between theory and practice and actively seek solutions. For instance, telemedicine education (12), virtual reality technology education (13), gamified education (14), and simulation-based teaching (15) have shown promising teaching outcomes to some extent. However, implementing these teaching models still faces challenges. Firstly, substantial investment in equipment and technological support is required. Secondly, adequate space and facilities are needed for simulation teaching. Additionally, a competent faculty is essential, with instructors possessing relevant skills and knowledge to effectively utilize teaching technologies and tools. Due to these constraints, the comprehensive implementation of these teaching models becomes challenging.

BOPPPS teaching mode also known as guided learning interactive additive education (16), was initially developed for teacher skill training to enhance teaching effectiveness and instructors’ teaching skills. It is a closed-loop instructional design model based on constructivism theory (17). The BOPPPS teaching model divides classroom instruction into six modules: Bridge-in, Objective, Pre-assessment, Participatory Learning, Post-assessment, and Summary. B (Bridge-in) is the introductory phase of classroom teaching where students’ attention is captivated through problem-based introduction, aiming to stimulate students’ interest in learning. O (Objective) refers to the achievable and assessable learning objectives set for students at different levels. P (Pre-assessment) is a diagnostic assessment conducted before the lesson to help teachers understand students’ current learning status and make necessary adjustments to the teaching content and pace. P (Participatory Learning) involves interactive and collaborative learning activities between teachers and students to achieve the learning objectives. P (Post-assessment) refers to the testing or evaluation of students at the end of the lesson to assess the effectiveness of teaching. S (Summary) involves summarizing the key points and concepts covered in the lesson (18).

In the past decade, there has been an increasing amount of research on BOPPPS in nursing education, including areas such as medical nursing (19), surgical nursing (20), health assessment (21), and nursing management (22). These studies indicate that the BOPPS teaching model has a positive impact on student’s academic performance, self-directed learning abilities, and satisfaction with their learning, compared to control groups receiving traditional teaching methods. In the application of the BOPPPS teaching model in nursing education, most of the studies have small sample sizes and lack RCTs. There is no high-level evidence comparing the instructional effectiveness of the BOPPPS teaching model to other teaching methods in nursing education. To understand the practical significance of this model in the foundational nursing courses, this systematic review aims to evaluate the impact of the BOPPPS teaching model on the effectiveness of teaching in “Fundamentals of Nursing” courses, including the theoretical score, practice score, self-learning ability score, and satisfaction rate of teaching effect.

## Methods

This systematic review and meta-analysis are conducted based on the Preferred Reporting Items for Systematic Reviews and Meta-Analyses (PRISMA) tool proposed by Page et al. (23). PRISMA is a set of evidence-based minimum items for reporting in systematic reviews and meta-analyses. The completed PRISMA checklist is provided as Supplementary material. Additionally, this review is conducted following the guidelines of the Cochrane Systematic Review and Intervention Manual but is not registered in a protocol (24).

### Search strategy

The literature search was conducted from January 2012 to September 14, 2023, using the following databases: PubMed, Web of Science, Embase, and the Cochrane Library. English keywords used for the search included BOPPPS (bridge-in, learning objective, pretest, participatory learning, posttest, and summary) and “Fundamentals of Nursing” education. Additionally, Chinese databases including CNKI, WanFang Data, and VIP were searched. Chinese keywords used for the search included BOPPPS, interactive additive education, and “Fundamentals of Nursing.” MeSH terms or titles or abstracts were used for the English database search, while subjects or titles or abstracts were used for the Chinese database search. The search strategies for different databases are provided as Supplementary material.

### Inclusion and exclusion criteria

#### Inclusion criteria

This study developed search strategies based on the PICOS criteria (Population, Intervention, Comparison, Outcome, and Study Design) (Table 1).

**Table 1:** Inclusion criteria based on PICO in this systematic review and meta-analysis.

Population	Students receiving “Fundamentals of Nursing” education in medical school
Intervention	BOPPPS teaching model
Comparison	Conventional Teaching Group
Outcomes	Primary: Theoretical score; Practice score
	Secondary: Self-learning ability score; Satisfaction rate of teaching effect
Study design	Randomized Clinical Trial (RCT)

#### Exclusion criteria

1. Observational studies or non-randomized controlled studies.
2. Not in the field of “Fundamentals of Nursing” education.
3. No reporting of quantitative outcomes.
4. Conference papers.
5. Non-Chinese articles, English articles.

### Data extraction

We extracted data from the studies based on the Cochrane Handbook for Systematic Reviews of Interventions (25). Two reviewers (YuL and YaL) independently screened the titles and abstracts of the included studies. Disagreements were resolved by discussion with a third researcher (YaL). The following information was extracted from each study: first author name, publication year, sample size, intervention measures, final scores, measurement time point, and learning outcomes.

### Risk of bias assessment

Two researchers independently assessed the quality of the included studies using the Cochrane Risk of Bias Tool (24). The evaluation criteria included the randomization process, deviation from intended interventions, missing outcome data, measurement of the outcome, and selection of the reported result. When there were disagreements, a third researcher was consulted for discussion. Each item was assessed in three categories: low risk, high risk, and unclear. If all the included literature items met the criteria for low risk (Grade A), some items met the criteria for low risk (Grade B), and each item failed to meet the criteria for low risk (Grade C), they were excluded. A funnel plot was employed to assess publication bias.

### Study quality

The included studies in this article are all RCTs. The quality of evidence for the outcome measures studied was further graded using the GRADE criteria (26). RCTs are considered the highest level of evidence, and the grading takes into account five factors: risk of bias in the studies, directness of the evidence, inconsistency of effect estimates (heterogeneity), precision of the effect estimates, and risk of publication bias. These factors are assessed to determine any downgrading or upgrading of the evidence quality.

### Statistical analysis

Meta-analysis was performed using Rev. Man 5.4 and Stata 17.0 software. For continuous data, such as theoretical score, practice score, and self-learning ability score, the standardized mean difference (MD) and corresponding 95% confidence interval (CI) were used to estimate the effect size. For categorical variables, such as the satisfaction rate of teaching effect, the relative risk (RR) and 95% CI were used for statistical evaluation. Heterogeneity tests were conducted for each study result. If *I*^2^ < 25%, it suggests low heterogeneity; if *I*^2^ = 25–75%, it suggests moderate heterogeneity; if *I*^2^ > 75%, it suggests high heterogeneity (27). If *I*^2^ < 50%, a fixed-effects model was used for analysis, indicating low to moderate heterogeneity or no statistical heterogeneity in the studies. If *I*^2^ ≥ 50%, a random-effects model was used for analysis.

## Results

### Search results

**Figure 1:**
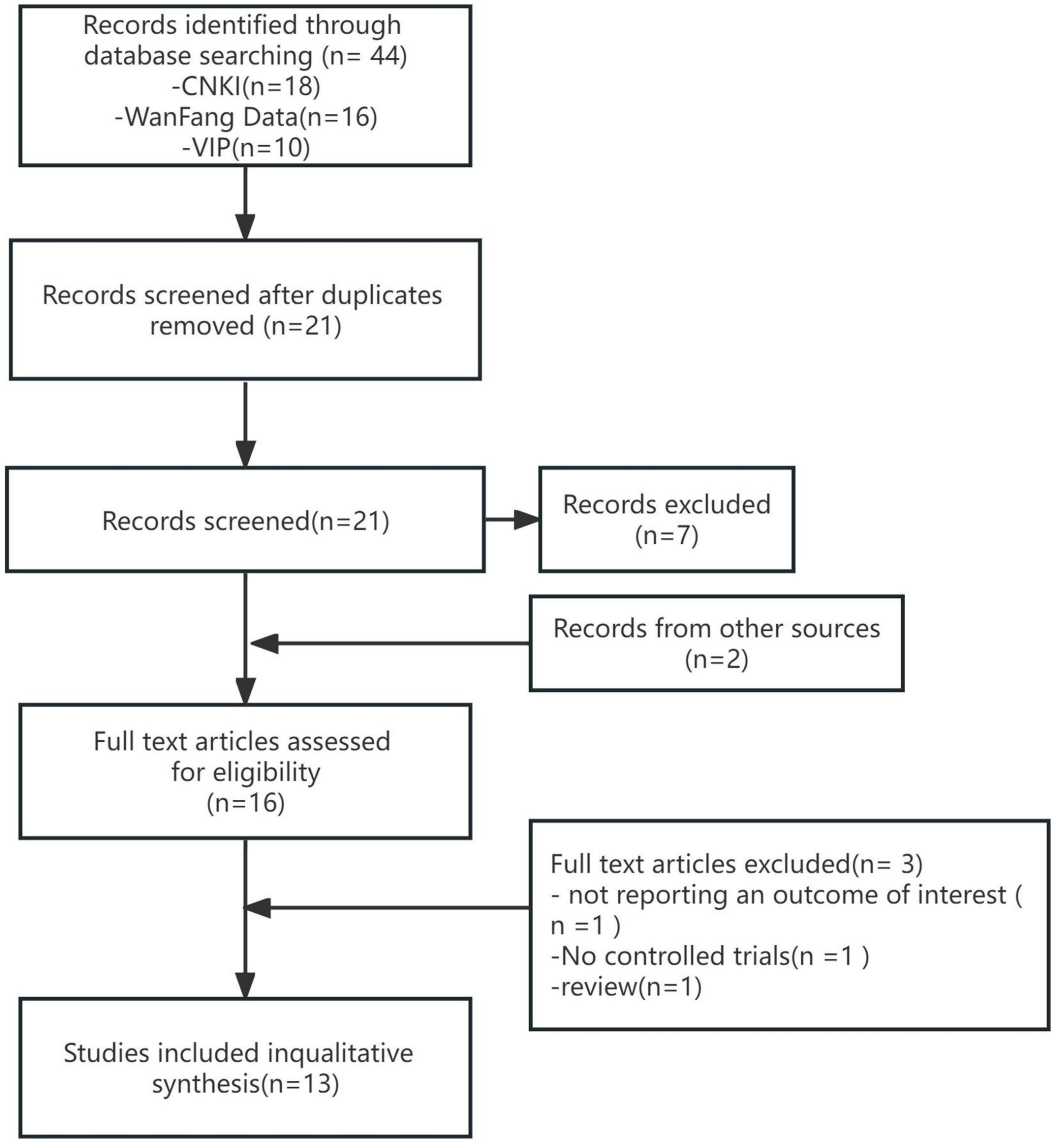
Flowchart of the search strategy.

A systematic search was conducted for both Chinese and English articles from January 2012 to September 14, 2023. The initial search yielded 44 relevant articles, and after removing 23 duplicate articles using Endnote software, 21 articles remained. Upon reviewing the titles and abstracts, 7 articles were excluded, leaving 14 articles. Through a snowballing technique, 2 additional articles were identified and included. A comprehensive review was conducted on the resulting 16 articles, leading to the exclusion of 3 articles due to insufficient data for extraction (*n* = 1), lack of controlled trials (*n* = 1), and being a review article (*n* = 1). Finally, based on inclusion and exclusion criteria, a total of 13 RCTs were included in this meta-analysis (Figure 1).

### General characteristics of the included studies

Table 2 presents the general characteristics of the included studies. All 13 included RCTs involved in the education of the “Fundamentals of Nursing” course, with a total of 2,991 nursing students participating in the experiments. Among them, 1,465 subjects received BOPPPS teaching in the experimental group, while 1,526 subjects received traditional teaching in the control group. All 13 articles included in the analysis were published in Chinese.

**Table 2:** General characteristics of the included studies (*n* = 10).

Author (year)	Sample size		Mode of teaching		Final test results		Measurement time point	Learning outcomes
	Intervention group	Control group	Intervention group	Control group	Intervention group	Control group		
Zhang et al. (28)	80	80	BOPPPS teaching mode	Traditional teaching method	78.99 ± 9.3	77.23 ± 8.78	Only after intervention	①②
Ni (29)	86	95	The teaching method of BOPPPS combined with PAD class	Routine teaching method	91.37 ± 5.56	86.90 ± 12.74	Baseline and after intervention	①②③④⑤
Bi (30)	67	67	BOPPPS teaching mode	Traditional teaching method	78.34	75.29	Only after intervention	①②
Hao et al. (31)	50	55	BOPPPS teaching based on ‘Xuexitong ‘platform	Traditional teaching method	86.83 ± 2.98	83.22 ± 1.39	Only after intervention	①③
Zhang et al. (32)	114	112	Based on ‘Rain Classroom ‘and BOPPPS model	Traditional teaching method	80.22 ± 7.88	77.39 ± 8.65	Baseline and after intervention	①②③
Liu et al. (33)	48	48	The teaching mode based on BOPPPS and PAD class	Routine teaching method	NA	NA	Baseline and after intervention	②③⑥
Wang (34)	106	109	BOPPPS teaching mode	Traditional teaching method	81.66 ± 6.35	79.61 ± 7.64	Baseline and after intervention	①②③④⑦
Xia et al. (35)	233	238	BOPPPS teaching mode	Traditional teaching method	87.67 ± 6.10	83.62 ± 8.97	Only after intervention	①⑧⑨
Liu et al. (36)	111	110	On the basis of conventional teaching, BOPPPS mold is carried out. Type of rain classroom teaching	Traditional teaching method	80.36 ± 7.77	77.96 ± 9.45	Only after intervention	①③
Zhou et al. (37)	57	60	BOPPPS teaching model based on Superstar Learning Platform	Traditional teaching method	78.67 ± 8.14	72.67 ± 12.07	Only after intervention	①③⑧
Li et al. (38)	261	259	BOPPPS teaching mode	Traditional teaching method	77.15 ± 7.03	75.40 ± 6.94	Only after intervention	①
Xu et al. (39)	143	187	BOPPPS teaching mode based on flipped classroom	Traditional teaching method	87.41 ± 4.58	85.37 ± 4.92	Only after intervention	①③⑩
Cui et al. (40)	109	106	BOPPPS teaching mode, with the help of the campus ‘real ‘teaching platform	Traditional teaching method	71.11 ± 0.75	62.64 ± 10.72	Only after intervention	①③

In the experimental group of the included studies, different learning platforms were used in combination with the BOPPPS teaching model. Among them, two studies used BOPPPS teaching approach combined with Presentation-Assimilation-Discussion (PAD) class teaching methods (29, 33), while five studies were based on the BOPPPS teaching model using information technology platforms (31, 32, 36, 37, 40). One study used a flipped classroom-based BOPPPS teaching model (39), and the remaining five studies used the BOPPPS teaching method without any additional intervention measures (28, 30, 34, 35, 38). The control group in all studies adopted traditional classroom teaching methods.

The meaning of the Learning outcomes column number is: ①course assessment results, students’ satisfaction with the teaching mode, ③self-learning ability, ④critical thinking ability, ⑤humanistic care ability, ⑥learning attitude, ⑦learning initiative, ⑧learning engagement level, ⑨learning adaptation level, ⑩Self-directed learning ability.

### Risk of bias in the included studies

**Figure 2:**
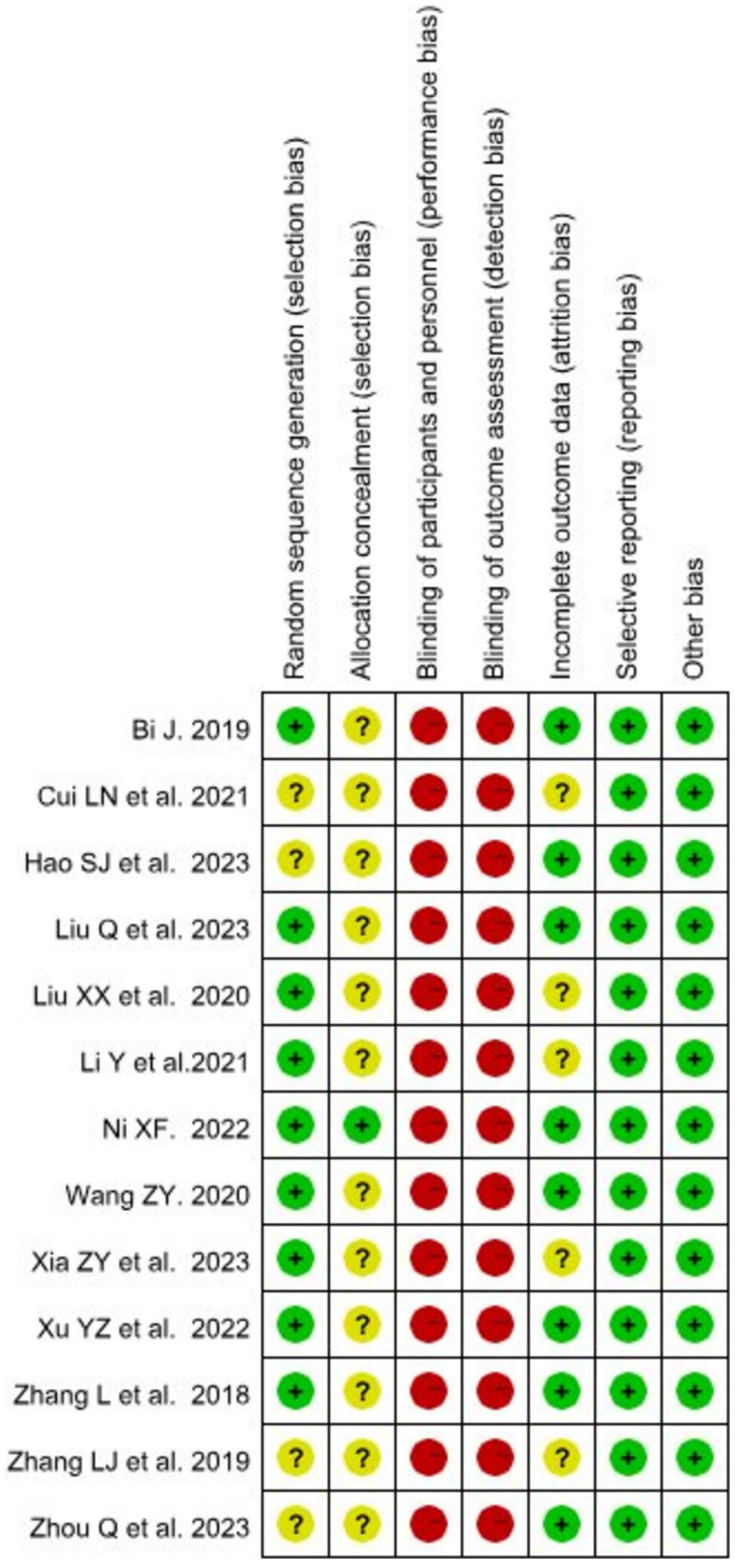
Risk of bias of included RCTs with the Cochrane RoB2 tool.

The results of the quality assessment for the included studies indicated that the overall risk of bias for each included study was considered “low risk of bias.” All included studies reported their outcome data, but 4 studies (30.7%) (31, 32, 37, 40) did not report the method of randomization, resulting in an assessment of “unclear.” Due to the possibility of participants and educators being aware of the assigned interventions during the study, 12 studies (92.3%) (28, 30–40) were deemed to have allocation bias, assessed as “unclear.” Given the nature of the interventions, blinding of students and teachers during the study was deemed impractical, resulting in a high-risk evaluation for participants and personnel blinding. Five studies (38.4%) (32, 35, 36, 38, 40) did not report attrition rates, resulting in an assessment of “unclear.” The quality assessment for the included studies (Figure 2).

### Data analysis

#### Theoretical score

**Figure 3:**
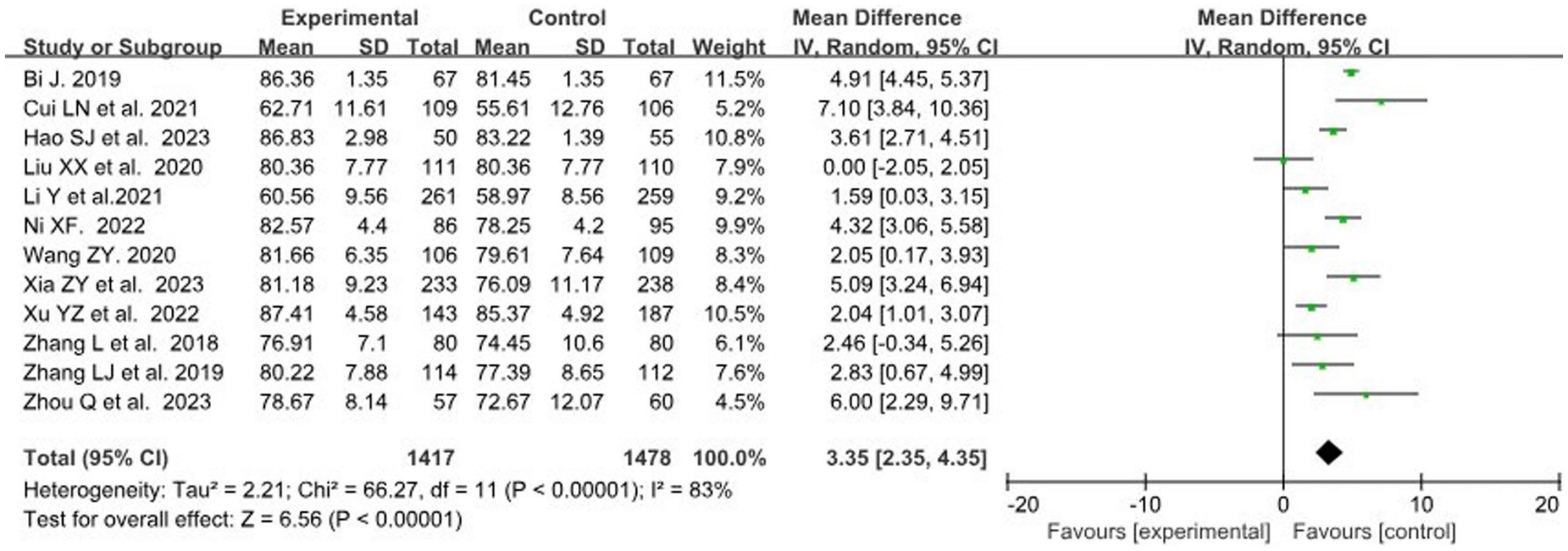
Forest plot of theoretical score for BOPPPS compared with the traditional teaching group.

A total of 12 studies (28–32, 34–40) with data related to Theoretical score were included, involving 1,417 and 1,478 students in the BOPPPS group and traditional teaching group, respectively. Compared with traditional teaching, the overall effect size of the 12 studies (MD = 3.35, 95% CI: 2.35–4.35, *Z* = 6.56, *p* < 0.00001) indicated a significant improvement in theoretical score in the BOPPPS teaching group. Due to significant statistical heterogeneity among the studies (*p* < 0.00001, *I*^2^ = 83% > 50%), a random-effects model was used for the meta-analysis (Figure 3).

#### Practice score

**Figure 4:**
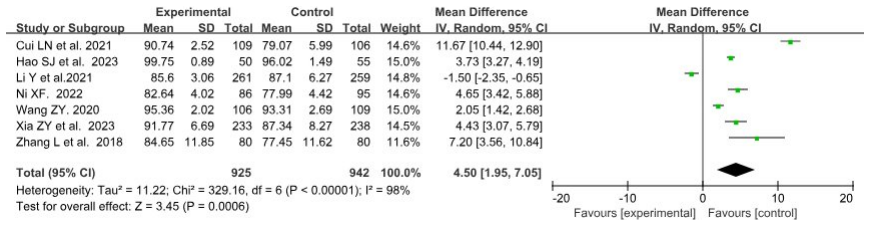
Forest plot of practice score for BOPPPS compared with the traditional teaching group.

A total of 7 studies (28, 29, 31, 34, 35, 38, 40) with data related to practice score were included, involving 925 and 942 students in the BOPPPS group and traditional teaching group, respectively. Compared with traditional teaching, the overall effect size of the 7 studies (MD = 4.50, 95% CI: 1.95–7.05, *Z* = 3.45, *p* = 0.0006) indicated a significant improvement in practice score in the BOPPPS teaching group. Due to significant statistical heterogeneity among the studies (*p* < 0.00001, *I*^2^ = 98% > 50%), a random-effects model was used for the meta-analysis (Figure 4).

#### Self-learning ability score

**Figure 5:**
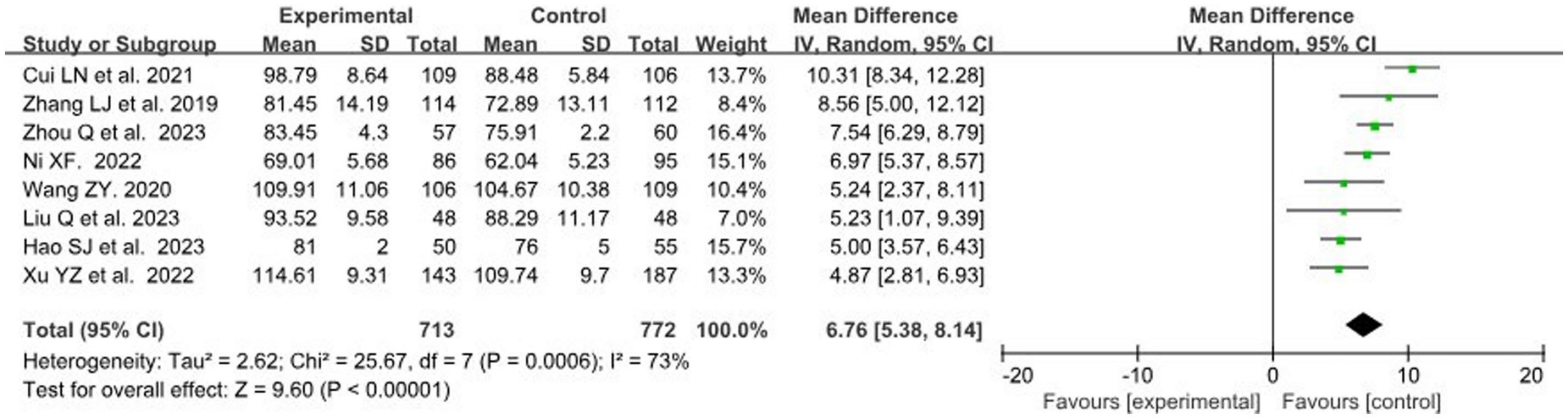
Forest plot of self-learning ability score for BOPPPS compared with the traditional teaching group.

A total of 8 studies (29, 31–34, 37, 39, 40) with data related to self-learning ability score were included, involving 824 and 882 students in the BOPPPS group and traditional teaching group, respectively. In this meta-analysis, the overall effect size of the 8 studies (MD = 6.76, 95% CI: 5.38–8.14, *Z* = 9.60, *p* < 0.00001) indicated a significantly higher self-learning ability score in the BOPPPS teaching group compared to the traditional teaching group. Due to moderate statistical heterogeneity among the studies (*p* = 0.0006, *I*^2^ = 73 > 50%), a random-effects model was used for the meta-analysis (Figure 5).

### Satisfaction rate of teaching effect

**Figure 6:**
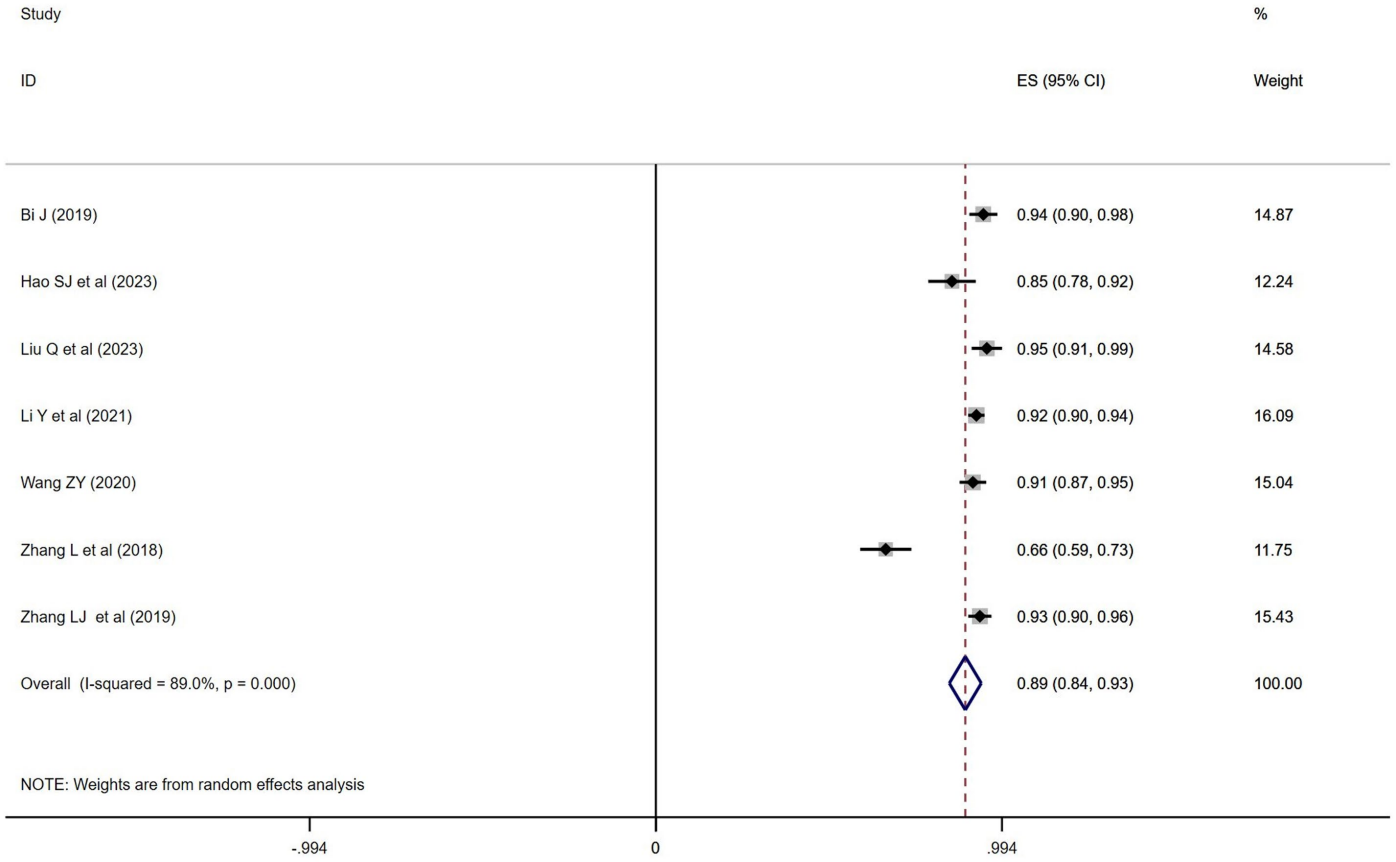
Meta-analysis of the satisfaction rate of teaching effect in BOPPPS group.

A total of 7 studies (28, 30–34, 38) related to the Satisfaction rate of teaching effect were included. Since only data on teaching satisfaction from the BOPPPS group were available in the included studies, a single proportion analysis was conducted using Stata 17.0 software. The results showed that the satisfaction rate of teaching among students in the BOPPPS group was 89% (95% CI = 0.84–0.93). Due to significant statistical heterogeneity among the studies (*p* = 0.000, *I*^2^ = 89% > 50%), a random-effects model was used for the meta-analysis (Figure 6).

### Publication bias

**Figure 7:**
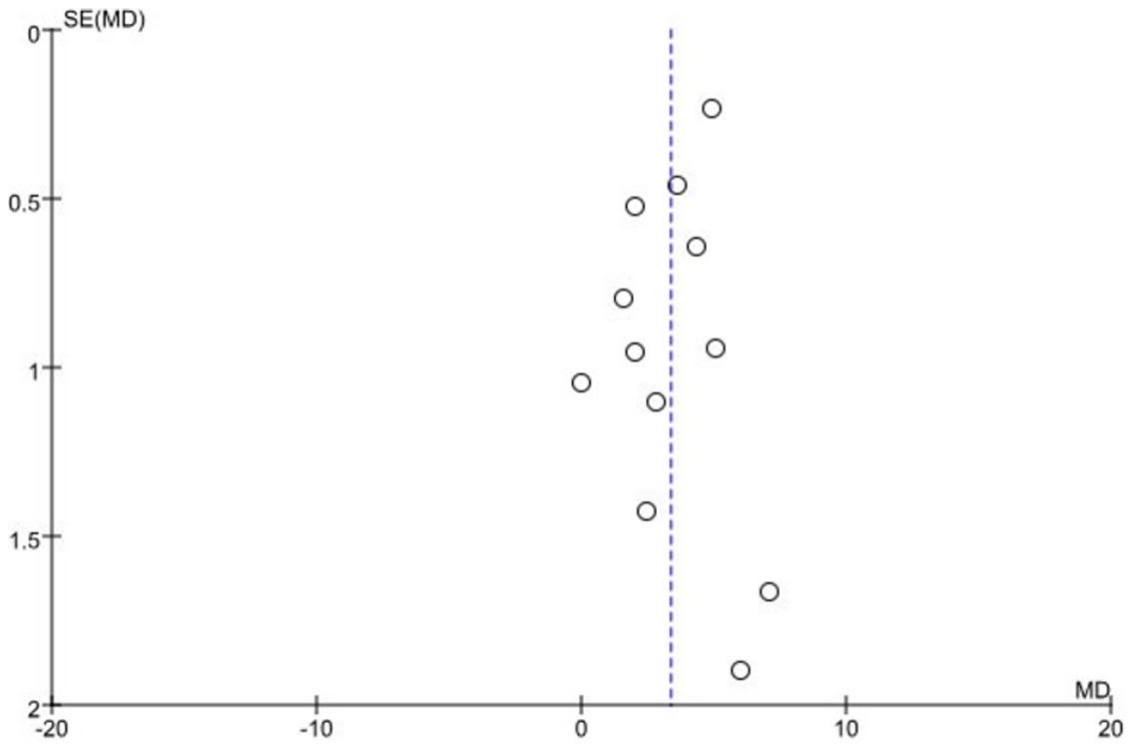
Funnel plot of theoretical score for BOPPPS compared with the traditional teaching group.

According to the Cochrane Handbook standards, fewer than 10 studies were included in the literature review on self-learning ability score, practice score, and satisfaction rate of teaching effect, making it impossible to conduct a funnel plot test for publication bias. Therefore, a funnel plot was created to assess publication bias for the theoretical score, which is a major objective evaluation indicator. The results showed that the funnel plot was generally symmetrical, with evenly distributed scatter points, indicating the credibility and reliability of the conclusions drawn from this study (Figure 7).

### Rating the quality of evidence

The included studies were RCTs, which represent the highest level of evidence. The GRADE evidence level for theoretical score and satisfaction rate of teaching effect was low. The evidence level for practice score was very low, and the evidence level for self-learning ability score was moderate. All studies reported outcome measures directly. The reasons for downgrading the evidence are as follows (Table 3).

**Table 3:** GRADE evidence summary table.

Outcomes	Quality assessment						Effect (95%CI)	No. of Participants (Studies)	Quality of the evidence (GRADE)
	Design	Risk of bias	Inconsistency	Indirectness	Imprecision	Other considerations			
Theoretical score	RCT	No serious risk of bias	Very serious^a^	No serious indirectness	No serious imprecision	None	SMD = 3.35,95%CI(2.35 ~ 4.35)	2,895 (12 studies)	ÅÅOO LOW
Practice score	RCT	No serious risk of bias	Very serious^a^	No serious indirectness	Serious^b^	None	SMD = 4.50,95%CI(1.95 ~ 7.05)	1867 (7 studies)	⊕ΟΟΟ VERY LOW
Self-learning ability score	RCT	No serious risk of bias	Serious^c^	No serious indirectness	No serious imprecision	None	SMD = 6.76,95%CI(5.38 ~ 8.14)	1,485 (8 studies)	⊕ ⊕ ⊕Ο MODERATE
Satisfaction rate of teaching effect	RCT	No serious risk of bias	Very serious^a^	No serious indirectness	No serious imprecision	None	RR = 0.89,95%CI(0.84 ~ 0.93)	1,456 (7 studies)	ÅÅOO LOW

CI, confidence interval; RCT, randomized trials. GRADE Working Group grades of evidence. High quality: Further research is very unlikely to change our confidence in the estimate of effect. Moderate quality: Further research is likely to have an important impact on our confidence in the estimate of effect and may change the estimate. Low quality: Further research is very likely to have an important impact on our confidence in the estimate of effect and is likely to change the estimate. Very low quality: We are very uncertain about the estimate.

a *I*^2^ > 75%.

b The overlap is not good.

c *I*^2^ > 50%.

## Discussion

Although the evidence level in the GRADE assessment may be relatively low, it still provides some evidence regarding the effectiveness and feasibility of the BOPPPS teaching method. Among these, randomized controlled trials represent the highest level of evidence, demonstrating that the included studies support the effectiveness of the BOPPPS method. This finding is crucial because randomized controlled trials are the most reliable and trustworthy method in research design.

Furthermore, evidence of moderate levels suggests that the Self-learning ability score provides some degree of support for the effectiveness of the BOPPPS teaching method. Despite the lower evidence levels for Theoretical score and Satisfaction rate of teaching effect, intervention studies show that BOPPPS can significantly improve these scores, with the BOPPPS group’s scores significantly higher than those of the control group. This indicates that BOPPPS may have certain benefits in terms of theoretical knowledge acquisition and teaching effectiveness, despite the exact evidence level being relatively low. Although the evidence level for practical scores is extremely low, intervention studies show that the scores of the BOPPPS group are significantly higher than those of the control group, suggesting that BOPPPS may have favorable effects in practical environments, although more research is needed to confirm this.

Although the issue of “inconsistency” exists in the GRADE assessment, it is necessary to consider factors such as the sample size involved in the study, the level of teachers in the research methods, curriculum construction, difficulty of assessment, and students’ learning abilities, which may influence the results.

In conclusion, despite the weak evidence level in the GRADE assessment, it can still be concluded based on the included studies, moderate evidence levels, and intervention effects that the BOPPPS teaching method has positive implications for nursing students’ Fundamentals of Nursing courses to some extent. However, more high-quality research is still needed to provide more compelling evidence to further support the effectiveness and feasibility of the BOPPPS method.

### The BOPPPS teaching model facilitates students’ comprehension of theoretical knowledge

The BOPPPS teaching model, through its comprehensive and organized structure, greatly facilitates students’ mastery of theoretical knowledge in nursing. This theoretical knowledge encompasses nursing science, technology, and theoretical frameworks in practice, such as understanding human anatomy and physiology, theories related to diseases and health, various models, and principles of nursing techniques (41). These form the cornerstone of professional growth for nursing students or nurses. The BOPPPS model emphasizes a student-centered teaching philosophy (42). By effectively connecting three stages: pre-class preview, in-class interactive participation, and post-class review, it provides students with a planned and systematic learning process (43). In the pre-class phase, through previewing, students can better prepare for and understand the upcoming content. During class, various teaching methods such as group discussions and scenario demonstrations not only stimulate students’ interest in learning but also deepen their understanding and mastery of knowledge. Additionally, classroom discussions and evaluation sessions enhance students’ learning motivation. Through questioning, problem-solving, and immediate feedback from teachers, students’ abilities and confidence in autonomous learning are enhanced. After class, by completing assignments and quizzes, students can self-assess their learning outcomes, and identify and fill in knowledge gaps, thus forming an effective learning cycle.

### The BOPPPS teaching model is advantageous in strengthening students’ proficiency in practical skills

The level of practical skills refers to the skill level and ability demonstrated by nursing students in nursing practice operations or actual clinical work (44). This includes but is not limited to, abilities in patient care procedures, correct usage of medical equipment, emergency treatment, patient observation, and evaluation of conditions (45). The level of practical skills directly impacts the quality of work for nursing professionals and the effectiveness of patient care (46). The BOPPPS teaching model is advantageous in facilitating the mastery of theoretical knowledge and enhancing skill levels. The pre-test and post-test of this teaching model serve to develop students’ independent thinking abilities while also enabling them to address any knowledge gaps they may have missed, thereby enhancing their comprehension and mastery of the subject (17). In class, students learn through discussions and actively engage in discussions as part of their learning process. The integration of these two aspects promotes a thorough understanding and solidification of knowledge for students (47). Building upon a solid theoretical framework, students delve into the intricacies of practical implementation through activities such as group discussions, presentations, and comprehensive evaluations. Moreover, having thoroughly examined the operational aspects and laid out initial procedures beforehand, students develop a high level of proficiency and the adaptability required to apply this knowledge across diverse clinical settings. Consequently, there is a seamless transition from theory to practice (48) yielding substantial improvements in their hands-on capabilities and analytical problem-solving aptitude. Ultimately, this translates into an elevated mastery of practical skills.

### The BOPPPS teaching model is advantageous in strengthening students’ ability for independent learning

The ability for self-directed learning refers to an individual’s capacity during the learning process to autonomously and actively engage in learning activities, including setting goals, developing study plans, acquiring information, problem-solving, reflection, and evaluation (49). This ability enables individuals to efficiently acquire the necessary knowledge and skills and to adapt and develop within constantly changing learning environments (50). Conventional teaching methods are limited in their ability to cultivate students’ self-directed learning skills, manifesting primarily in insufficient motivation levels (19). Typically, students rely on educational management from their schools and teachers, thereby lacking a sense of accountability and exhibiting poor self-control and self-management strategies (51). By comparison, control groups tend to adopt a passive approach to learning and often struggle with boredom, leading to low motivation and minimal levels of initiative during the learning process. The BOPPPS teaching model is rooted in promoting students’ independence (52). It places great emphasis on students autonomously obtaining pertinent learning information through various means, including pre-class readings, in-class group discussions, and post-class reinforcement activities (53). As students engage in this process, their capabilities in information acquisition and processing steadily improve, leading to enhanced self-management skills and a growing aptitude for self-directed learning.

### The BOPPPS teaching model is advantageous in enhancing students’ satisfaction with the effectiveness of teaching

Student satisfaction rate with teaching refers to the ratio or percentage of the overall satisfaction level of students with the teaching process. This data is typically obtained through surveys or evaluations of students. The level of student satisfaction with teaching reflects the proportion of their approval and satisfaction with the teaching program (54). The BOPPPS teaching model emphasizes equality between teachers and students, with the latter serving as the main agents of learning (55). It promotes self-directed learning, thereby enabling students to transform from passive learners to active ones. Through class activities that encourage student-to-student and teacher-to-student interactions, the traditional teaching approaches have been disrupted, creating a stimulating and energetic classroom environment (56). Such an atmosphere enhances students’ participation in educational activities, facilitating the development of their overall skills, and elevating their interest and productivity levels. Students value classes filled with interactive and engaging content, showing a willingness to actively participate in educational endeavors, thereby elevating their satisfaction with the quality of teaching.

### Limitations of BOPPPS teaching mode in fundamentals of nursing education

The BOPPPS model has the following limitations: (1) Despite its widespread application in the field of education, there is relatively little specific research on its effectiveness in Fundamentals of Nursing education, and a lack of sufficient empirical studies may restrict understanding of its actual impact in nursing curricula. (2) The inclusion of studies with short implementation periods and small sample sizes may affect understanding of whether this teaching model is suitable for widespread adoption in nursing education. (3) The heterogeneous nature of the BOPPPS intervention measures used in studies may make comparative results difficult. (4) Effective implementation of the BOPPPS method may require teachers to undergo appropriate training. However, the methods and levels of nursing teacher training included in the studies vary, which may affect the results and lead to heterogeneity. (5) The BOPPPS method may consume more resources than traditional teaching methods, as it requires additional planning, preparation, and teacher support.

### Limitations of research and analysis

The analysis presented in this article has some limitations. (1) The studies ultimately included in the analysis were all conducted in China, with participants consisting solely of Chinese students. This may reduce the representativeness and generalizability of the results. (2) Based on the scientific nature of the study, researchers should strive to minimize implementation bias in quasi-experimental research to accurately determine the impact of teaching models on student learning outcomes. However, implementing blinding procedures for research participants during the teaching process is impractical because the instructional mode employed cannot be concealed from the students. Therefore, blinding the students presents significant challenges. (3) There is significant heterogeneity among the studies included in this analysis, which may be attributed to factors such as differences in teaching qualifications, course design, assessment difficulty, and variation in learning abilities. This poses a challenge to conducting a systematic meta-analysis. (4) The quality of a meta-analysis relies on the quality of the data from included studies. As the quality of the included literature is relatively low and the sample sizes are limited, there is a need for more publications and the inclusion of large-scale, multicenter, and high-quality studies. (4) There is currently no standard guideline for the application of BOPPPS in medical disciplines in China, nor are there effective criteria for evaluating the BOPPPS teaching model.

## Author contributions

YuL: Data curation, Investigation, Writing – original draft, Writing – review & editing. XL: Supervision, Validation, Writing – review & editing. YnL: Investigation, Methodology, Writing – original draft. YgL: Investigation, Methodology, Resources, Writing – review & editing.
