# Supplementary material for: Application effect of BOPPPS teaching model on fundamentals of nursing education: a meta-analysis of randomized controlled studies
Source: Front Med (Lausanne). 2024 May 9;11:1319711. doi: 10.3389/fmed.2024.1319711 (PMC11111886; doi:10.3389/fmed.2024.1319711)
Supplement: Supplementary file 1 [file Data_Sheet_1.ZIP › Supplementary material/S1_PRISMA_2020_checklist.docx]

| **Section and Topic** | **Item #** | **Checklist item** | **Location where item is reported** |
| --- | --- | --- | --- |
| **TITLE** | | |  |
| Title | 1 | Identify the report as a systematic review. | 1 |
| **ABSTRACT** | | |  |
| Abstract | 2 | See the PRISMA 2020 for Abstracts checklist. | 1 |
| **INTRODUCTION** | | |  |
| Rationale | 3 | Describe the rationale for the review in the context of existing knowledge. | 2-3 |
| Objectives | 4 | Provide an explicit statement of the objective(s) or question(s) the review addresses. | 2-3 |
| **METHODS** | | |  |
| Eligibility criteria | 5 | The specified research features ( PICOS ) and report features ( such as retrieval time, retrieved courses, language, and publication status ) were used as criteria for inclusion in the study and explanations were given. | 4 |
| Information sources | 6 | Describe the source of all the literature information for each search and the final search results. | 4 |
| Search strategy | 7 | Develop search strategies based on PICOS criteria ( population, intervention, control, results, and study design ). | 4 |
| Selection process | 8 | Two researchers identified and selected data in detail and independently from the study, and these differences were resolved by consensus or collaboration with a third member of the research team. |  |
| Data collection process | 9 | Two researchers extracted data from the study in detail, and the differences were resolved by consensus or in collaboration with a third member of the research team. | 4 |
| Data items | 10a | Results indicators to be collected, first author 's name, publication year, sample size, interventions, final grade, measurement time point and learning outcomes are described in detail. Main outcome measures : theoretical achievement, practical achievement ) ; secondary outcome measures : Self-learning ability score ; satisfaction rate of teaching effect. | 4 |
|  | 10b | .participant : students receiving basic nursing education ; interventions : education model based on BOPPPS | 4 |
| Study risk of bias assessment | 11 | Independently assess the quality of included studies using the Cochrane risk of bias tool | 4-5 |
| Effect measures | 12 | For continuous data : Theoretical score, Practice score, Self-learning ability score, we use standardized mean difference ( MD ) and related 95 % confidence interval ( CI ) to estimate the effect size. For the categorical variable : Satisfaction rate of teaching effect, we used relative risk ( RR ) and 95 % confidence interval ( CI ) for statistical evaluation. | 5 |
| Synthesis methods | 13a | Describe the processes used to decide which studies were eligible for each synthesis (e.g. tabulating the study intervention characteristics and comparing against the planned groups for each synthesis (item #5)). | 4 |
|  | 13b | Describe any methods required to prepare the data for presentation or synthesis, such as handling of missing summary statistics, or data conversions. | 4 |
|  | 13c | Use Excel for data statistics | 4 |
|  | 13d | Meta-analysis was performed using RevMan 5.4 and Stata 17.0 software. If I^2^ < 25%, it suggests low heterogeneity; if I^2^ = 25%-75%, it suggests moderate heterogeneity; if I^2^ > 75%, it suggests high heterogeneity [23]. If I^2^ < 50%, a fixed-effects model was used for analysis, indicating low to moderate heterogeneity or no statistical heterogeneity in the studies. If I^2^ ≥ 50%, a random-effects model was used for analysis. | 5 |
|  | 13e | Descriptive analysis was used to analyze the heterogeneity between the research results. | 5 |
|  | 13f | If I^2^ < 50%, a fixed-effects model was used for analysis, indicating low to moderate heterogeneity or no statistical heterogeneity in the studies. If I^2^ ≥ 50%, a random-effects model was used for analysis. | 5 |
| Reporting bias assessment | 14 | Two researchers independently assessed the quality of the included studies using the Cochrane Risk of Bias Tool. | 4 |
| Certainty assessment | 15 | The evidence quality of the outcome indicators was further rated using the evidence grading recommendation standard GRADE. | 5 |
| **RESULTS** | | |  |
| Study selection | 16a | Describe the results of the search and selection process, from the number of records identified in the search to the number of studies included in the review, ideally using a flow diagram. | 5-6 |
|  | 16b | Cite studies that might appear to meet the inclusion criteria, but which were excluded, and explain why they were excluded. | 5-6 |
| Study characteristics | 17 | Table 2 shows the characteristics of each study extracted. | 7-8 |
| Risk of bias in studies | 18 | The quality grade evaluation of the included studies ( Fig.2 ). | 7-8 |
| Results of individual studies | 19 | Meta-analysis was performed on the outcome indicators : theoretical score ( Figure 3 ), practice score ( Figure 4 ), self-learning ability score ( Figure 5 ) and satisfaction rate of teaching effect ( Figure 6 ). | 9-10 |
| Results of syntheses | 20a | The publication bias of Theoretical score was tested by making a funnel plot. The results show that the left and right sides of the funnel plot are basically symmetrical, and the scatter points are evenly distributed. | 12 |
|  | 20b | BOPPPS teaching model had significantly higher scores in theoretical score (MD=3.35, 95% CI: 2.35-4.35, Z = 6.56, P < 0.00001), practice score (MD=4.50, 95% CI: 1.95-7.05, Z = 3.45, P = 0.0006), and self-learning ability score (MD=6.76, 95% CI: 5.38-8.14, Z = 9.60, P < 0.00001) compared to the traditional teaching group. The satisfaction rate of students in the BOPPPS teaching group regarding the teaching effectiveness was 89% (95% CI=0.84-0.93).The differences were statistically significant (P < 0.05). | 10-11 |
|  | 20c | The heterogeneity between studies was descriptively analyzed in the limitations of the study. | 14 |
|  | 20d | If I^2^ < 50%, a fixed-effects model was used for analysis, indicating low to moderate heterogeneity or no statistical heterogeneity in the studies. If I^2^ ≥ 50%, a random-effects model was used for analysis. | 10-11 |
| Reporting biases | 21 | Risk of bias in the included studies | 8 |
| Certainty of evidence | 22 | GRADE evidence summary table | 12 |
| **DISCUSSION** | | |  |
| Discussion | 23a | Provide a general interpretation of the results in the context of other evidence. | 13-15 |
|  | 23b | Discuss any limitations of the evidence included in the review. | 14-15 |
|  | 23c | Discuss any limitations of the review processes used. | 14-15 |
|  | 23d | Discuss implications of the results for practice, policy, and future research. | 15 |
| **OTHER INFORMATION** | | |  |
| Registration and protocol | 24a | Not applicable. | 18 |
|  | 24b | No agreement prepared | 3 |
|  | 24c | No agreement prepared | 3 |
| Support | 25 | The authors declare that they have no source of funding for the research. | 18 |
| Competing interests | 26 | The authors declare that they have no competing interests. | 19 |
| Availability of data, code and other materials | 27 | The data included in the study and the data used for analysis are provided in the annex. | 19 |

*From:*  Page MJ, McKenzie JE, Bossuyt PM, Boutron I, Hoffmann TC, Mulrow CD, et al. The PRISMA 2020 statement: an updated guideline for reporting systematic reviews. BMJ 2021;372:n71. doi: 10.1136/bmj.n71

For more information, visit: <http://www.prisma-statement.org/>
