# Supplementary material for: Application effect of BOPPPS teaching model on fundamentals of nursing education: a meta-analysis of randomized controlled studies
Source: Front Med (Lausanne). 2024 May 9;11:1319711. doi: 10.3389/fmed.2024.1319711 (PMC11111886; doi:10.3389/fmed.2024.1319711)
Supplement: Supplementary file 1 [file Data_Sheet_1.ZIP › Supplementary material/S2_Table.docx.docx]

**S 2 Search strategy**

| Database | Search Details | Results |
| --- | --- | --- |
| PubMed | ("BOPPPS"[Title/Abstract] OR (("bridge-in"[All Fields] AND ("goals"[MeSH Terms] OR "goals"[All Fields] OR "objective"[All Fields] OR "objectives"[All Fields] OR "objectively"[All Fields]) AND "pre-assessment"[All Fields] AND "participatory"[All Fields] AND ("learning"[MeSH Terms] OR "learning"[All Fields] OR "learn"[All Fields] OR "learned"[All Fields] OR "learning s"[All Fields] OR "learnings"[All Fields] OR "learns"[All Fields]) AND "post-assessment"[All Fields]) AND "summary"[Title/Abstract])) AND "fundamentals nursing"[Title/Abstract] | 0 |
| web of science | ((TS=(BOPPPS)) OR TS=(bridge-in, objective, pre-assessment, participatory learning, post-assessment, summary)) AND TS=(Fundamentals of Nursing) | 0 |
| Embase | #5 #3 AND #4 0  #4 fundamentals AND of AND nursing 1256  #3 #1 OR #2 16  #2 'bridge in,' AND objective, AND 'pre assessment,' AND participatory AND learning, AND 'post assessment,' AND summary 1  #1 boppps 16 | 0 |
| cochrane | #1 BOPPPS 3  #2 bridge-in, objective, pre-assessment, participatory learning, post-assessment, summary 0  #3 #1 OR #2 3  #4 Fundamentals of Nursing 98  #5 #3 and #4 0 | 0 |
| CNKI,WanFang Data, VIP databases | (主题: BOPPPS(精确) OR (主题:导学互动的加式教育(精确AND( (主题: 护理学基础(精确)) OR (主题: 基础护理学(精确) ) | CNKI:18  WanFang Data:16  VIP databases:10 |
